# The Pharmaceutical Industry in 2025: An Analysis of FDA Drug Approvals from the Perspective of Molecules

**DOI:** 10.3390/molecules31030419

**Published:** 2026-01-26

**Authors:** Beatriz G. de la Torre, Fernando Albericio

**Affiliations:** 1 School of Laboratory Medicine and Medical Sciences, College of Health Sciences, University of KwaZulu-Natal, Durban 4001, South Africa; 2 School of Chemistry and Physics, University of KwaZulu-Natal, Durban 4001, South Africa; 3 Department of Inorganic and Organic Chemistry, University of Barcelona, 08028 Barcelona, Spain

**Keywords:** ADC, antibodies, antibody drug conjugate, aromatic-based drugs, biologics, chemical entities, fluorine-based drugs, natural products, new chemical entities, oligonucleotides, PFAS, peptides, polyfluoroalkyl substances, TIDES

## Abstract

In 2025, the U.S. Food and Drug Administration (FDA) approved 44 new drugs, reflecting a slight decrease compared to previous years but maintaining the overall trends in pharmaceutical innovation. Biologics accounted for 25% of approvals, including nine monoclonal antibodies (mAbs), two antibody–drug conjugates (ADCs), and one fusion protein, with cancer remaining the primary therapeutic focus. TIDES, comprising three oligonucleotides and one peptide, continued to consolidate their presence in the market, with the three oligonucleotides featuring N-acetylgalactosamine (GalNAc) for liver-targeted delivery. Small molecules dominate the remainder, with a high prevalence of N-aromatic moieties and fluorine atoms present in most of the molecules. Peptide manufacturing and sustainability concerns, including PFAS usage, remain key challenges. Despite these advances, the high cost of innovative therapies limits access, particularly in low- and middle-income countries. This report provides a structural and chemical analysis of the newly approved drugs, highlighting trends in molecular design, therapeutic areas, and technological innovations shaping modern drug discovery.

## 1. Analysis

**Figure 1:**
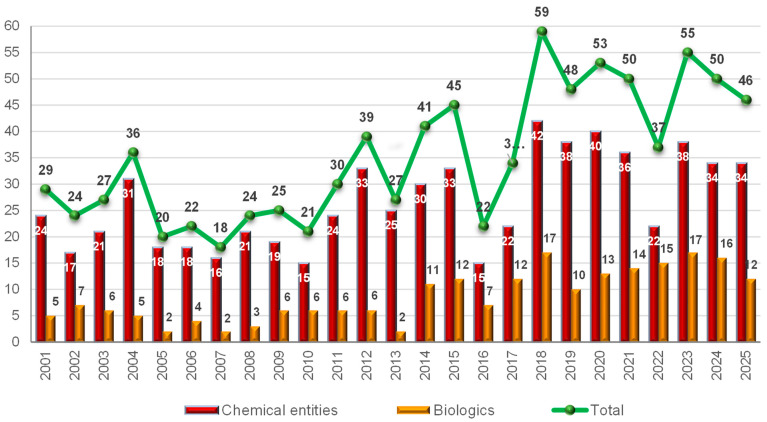
Drugs (new chemical entities and biologics) approved by the FDA in recent years^.^ Adapted with permission from ref. [1]. Copyright 2025 copyright MDPI [1,2].

An analysis of the number of new drugs approved by the U.S. Food and Drug Administration (FDA) during this century (Figure 1) shows that, during the first decade (2000–2010), an average of 25 drugs were approved per year. This number increased to 34 per year during the period 2011–2017 and further rose to approximately 50 per year between 2018 and 2024 [1]. In 2025, the FDA approved 46 new drugs [2], a figure slightly lower than in recent years. These results do not appear to be directly associated with the performance of the pharmaceutical industry itself, but rather with systemic issues related to the new U.S. administration, which has placed the pharmaceutical sector in the eye of the storm.

In 2025, the 46 newly approved drugs are divided into 34 new chemical entities—including TIDES (peptides and oligonucleotides)—and 12 biologics, comprising monoclonal antibodies (mAbs), antibody–drug conjugates (ADCs), and proteins (Figure 1). The number of biologics approved is lower than the average observed over the previous seven years (an average of 14.5 new biologics per year between 2018 and 2024 [1]. This decline is evident both in absolute and relative terms. During that period, biologics accounted for 29% of all approved drugs (102 out of 352), whereas in 2025 they represent 26% (12 out of 46) of the total.

After a two-year hiatus without new ADC approvals, two ADCs were approved in 2025. Among TIDES, four new drugs reached the market this year, representing nearly 10% of total approvals. Of these, three are oligonucleotides, and one is a peptide, further confirming the strong and sustained presence of oligonucleotides in the pharmaceutical landscape.

The Center for Biologics Evaluation and Research (CBER) approved nine Biologics License Applications (BLAs) in 2025 [3], a number comparable to those approved in 2021 and 2022, when 13 and 12 BLAs were registered, respectively. This figure is substantially lower than the 22 and 24 approvals recorded in 2023 and 2024, respectively. Among the approved biologics are two COVID-19 vaccines (from ModernaTX, Inc. and Novavax, Inc.), as well as vaccines for chikungunya (Bavarian Nordic A/S) and meningococcal disease (GlaxoSmithKline Biologicals) [3]. The importance of vaccines for societal well-being remains evident, despite the presence of some opposing views.

As in previous articles in this series, this work analyzes the 46 drugs approved in 2025 from a strictly structural chemical perspective. To enhance readability, the number of references has been intentionally kept to a minimum. Ref. [2] provides more information about those 44 new drugs.

## 2. Discussion

Table 1 summarizes the 12 biologics approved by the FDA in 2025, along with their corresponding indications. Although this total is slightly lower than in recent years (with 17, 10, 13, 14, 15, 17, and 16 approvals between 2018 and 2024), it remains in the double digits and is consistent with historical trends. Of the 2025 approvals, nine are mAbs, one in a combination with a protein; two are ADCs; and one is a fusion protein. Notably, two ADCs were approved this year, in contrast to the absence of ADC approvals in the preceding two years. As a result, the cumulative number of FDA-approved ADCs has reached 16 to date.

mAbs continue to be the predominant class of drugs. More than 90% of approved biologics and nearly 25% of all FDA-approved drugs are or contain mAb components. Cancer remains the primary indication for mAbs; however, mAb-based therapies are increasingly being applied to other disease areas.

Notably, penpulimab-kcqx was first approved in China in 2021 as a third-line chemotherapy for the treatment of adults with relapsed or refractory classical Hodgkin lymphoma. In the United States, it has been approved for the treatment of recurrent or metastatic nasopharyngeal carcinoma (NPC), either as monotherapy or in combination with cisplatin or carboplatin plus gemcitabine.

Keytruda Qlex^TM^ is a combination product comprising the monoclonal antibody pembrolizumab, a programmed death receptor-1 (PD-1)–blocking antibody, and berahyaluronidase alfa, a glycosylated hyaluronidase variant. Pembrolizumab was first approved by the FDA in 2014 for melanoma and subsequently for multiple other cancer types. Berahyaluronidase alfa facilitates the delivery of pembrolizumab.

Among mAb–based therapies that do not target cancer, Andembry^TM^ is indicated for the treatment of hereditary angioedema (HAE). In 2025, three therapies were approved for this indication; in addition to Andembry^TM^, the oligonucleotide Dawnzera^TM^ (donidalorsen) and the small molecule Ekterly^TM^ (sebetralstat) were also registered for the treatment of HAE (see below). HAE is a rare genetic disorder characterized by recurrent episodes of swelling affecting the face, extremities, gastrointestinal tract, and upper airway, the latter posing a potentially life-threatening risk of suffocation. Imaavy^TM^ (nipocalimab) is for the prevention of recurrent attacks in patients with the autoimmune disease myasthenia gravis. Enflonsia^TM^ (clesrovimab-cforis) is approved for the prevention of respiratory tract disease caused by RSV in neonates and infants born during the RSV season. Voyxact^TM^ (sibeprenlimab-szsiis) is indicated to reduce proteinuria in adults with primary immunoglobulin A (IgA) nephropathy. which is not a disease itself but rather a symptom resulting from an abnormal amount of albumin leaking from the blood into the urine, potentially leading to kidney damage. Vanrafia^TM^ (atrasentan), a small molecule, was also approved for the reduction of proteinuria (see below). Yartemlea^TM^ (narsoplimab-wuug) was a mAb approved for the treatment of thrombotic microangiopathy associated with hematopoietic stem cell transplantation.

Lerochol^TM^ is a third-generation proprotein convertase subtilisin/kexin type 9 (PCSK9) inhibitor [following Praluent^TM^ (alirocumab) and Repatha^TM^ (evolocumab)] designed to reduce low-density lipoprotein cholesterol (LDL-C), commonly referred to as “bad cholesterol.” Together with Leqvio^TM^ (inclisiran), a small interfering ribonucleic acid (siRNA)-based therapy approved in 2021, Lerochol^TM^ represents one of the newest therapeutic options for managing hypercholesterolemia, a major risk factor for cardiovascular disease.

**Figure 2:**
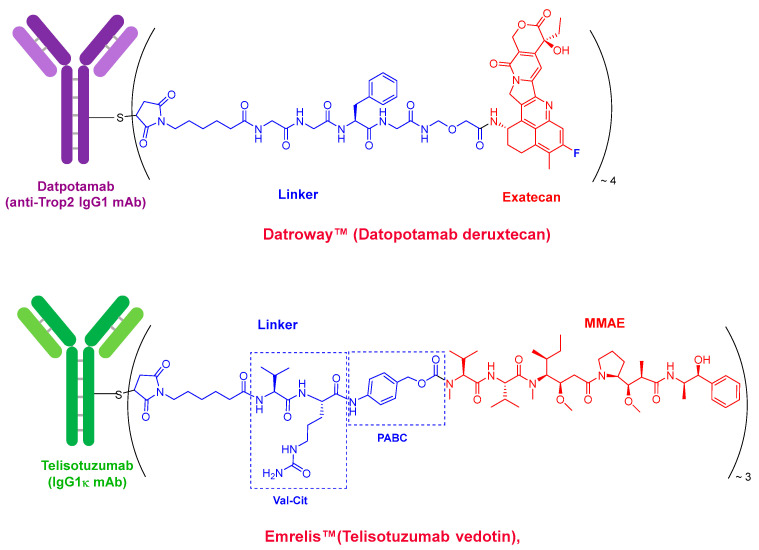
Structure of Datroway^TM^ (Datopotamab deruxtecan) and Emrelis^TM^ (Telisotuzumab vedotin).

Datroway^TM^ (Datopotamab deruxtecan) and Emrelis^TM^ (Telisotuzumab vedotin) (Figure 2), the two ADCs approved this year, follow the established paradigm of ADCs for cancer treatment. Both clearly exemplify this class of therapeutics. Datroway^TM^ contains an exatecan derivative, a fluorinated topoisomerase I inhibitor, as a cytotoxic payload. This is also present in Enhertu^TM^ (Trastuzumab deruxtecan), approved by the FDA in 2019, and belongs to the same family as govitecan, the payload in Trodelvy^TM^ (Sacituzumab govitecan), approved by the FDA in 2020.

In contrast, Emrelis^TM^ employs vedotin as its payload. Vedotin is a derivative of monomethyl auristatin E (MMAE), a synthetic peptide originally derived from dolastatins isolated from marine shell-less mollusks. Vedotin is also used in several other ADCs, including Adcetris^TM^ (Brentuximab vedotin, first approved by the FDA in 2011), Polivy^TM^ (Polatuzumab vedotin, approved in 2019), Padcev^TM^ (Enfortumab vedotin, approved in 2019), and Tivdak^TM^ (Tisotumab vedotin, approved in 2021), and is from the same payload family as mafodotin, which is present in Blenrep^TM^ (Belantamab mafodotin, approved in 2020). Peptide derivatives of MMAE represent the most commonly used payloads in ADCs.

Both Datroway^TM^ and Emrelis^TM^ incorporate a maleimide moiety to conjugate the payload to the antibody via cysteine residues and utilize cleavable linkers. Datroway^TM^ contains a tetrapeptide (Gly–Gly–Phe–Gly) that is cleaved by tumor-associated enzymes such as cathepsins. Emrelis^TM^ uses a self-immolative linker based on the Val–Cit dipeptide, which is also cleavable by cathepsins. In both cases, the cleavage takes place inside of the cell.

**Figure 3:**
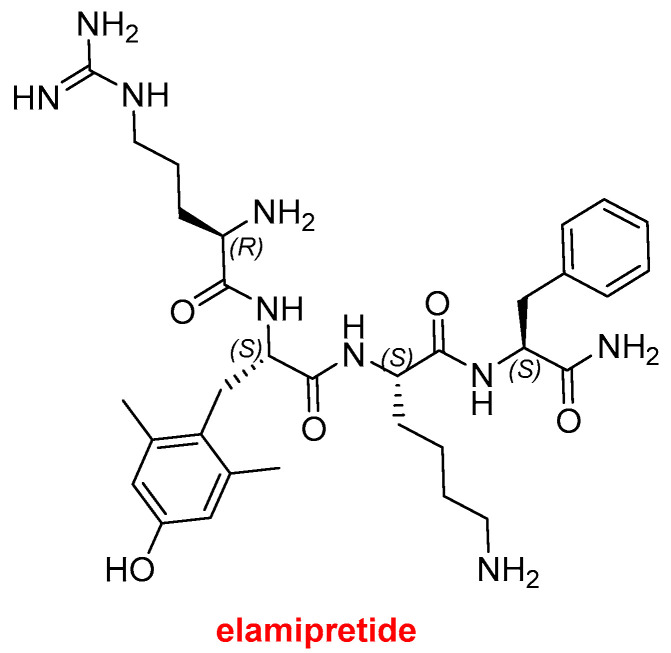
Structure of Forzinity^TM^ (elamipretide).

One peptide was approved by the FDA in 2025, bringing the total number of peptides on the market to approximately 130 [4]. Forzinity^TM^ (elamipretide) (Figure 3) is a small linear peptide with a C-terminal amidation, containing a D-Arg residue and a non-canonical dimethyltyrosine (H-D-Arg-2,6-diMe-L-Tyr-L-Lys-L-Phe-NH_2_). Forzinity^TM^ represents the first therapy approved to improve muscle strength in patients with Barth syndrome (BTHS), a rare X-linked genetic disorder that primarily affects males. BTHS manifests as skeletal muscle weakness, neutropenia, cardiomyopathy, profound fatigue or exercise intolerance, delayed growth and development, and often leads to premature death. Beyond BTHS, Forzinity^TM^ is being investigated for additional indications, including heart failure, hypertension, enhanced wound healing, and macular degeneration.

**Figure 4:**
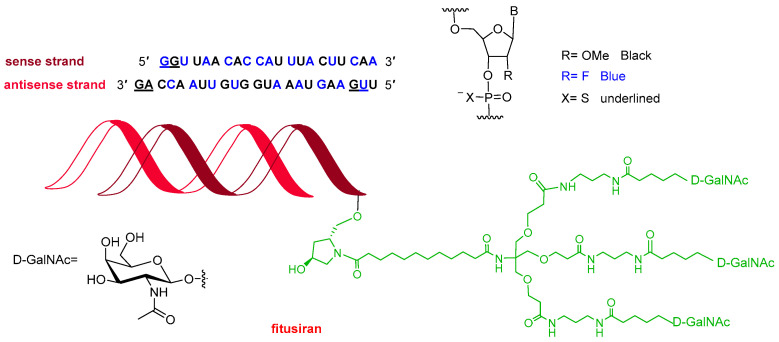
Structure of Qfitlia^TM^ (fitusiran).

Following recent trends, 2025 saw the approval of three oligonucleotides, bringing the total number of these molecules on the market to 24. Qfitlia^TM^ (fitusiran) (Figure 4) is a double-stranded siRNA indicated for the prevention or reduction of bleeding episodes in patients with hemophilia A or B. It acts by lowering levels of antithrombin, a small glycoprotein that inhibits enzymes in the coagulation cascade. Both strands of Qfitlia^TM^ are extensively chemically modified to enhance stability, including 21 2′-fluoro (2′-F) substitutions, 23 2′-O-methyl (2′-OMe) substitutions, and six phosphorothioate (PS) linkages at the strand termini. In addition, the siRNA is conjugated to a triantennary N-acetylgalactosamine (GalNAc) ligand to facilitate targeted delivery to hepatocytes.

**Figure 5:**
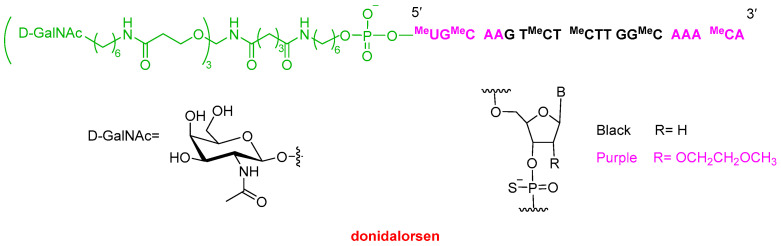
Structure of Dawnzera^TM^ (donidalorsen).

The second oligonucleotide approved this year, Dawnzera^TM^ (donidalorsen) (Figure 5), is indicated for the treatment of HAE, the same indication as the mAb Andembry^TM^ (garadacimab-gxii) discussed above and the small molecule Ekterly^TM^ (sebetralstat) (see below). Dawnzera^TM^ is an antisense oligonucleotide (ASO) conjugated to a triantennary N-acetylgalactosamine (GalNAc) ligand, enabling targeted delivery to hepatocytes, similar to Qfitlia^TM^ and most oligonucleotides approved in recent years. Like Qfitlia^TM^ and other marketed oligonucleotides, Dawnzera^TM^ incorporates chemically modified nucleotides to enhance stability. Specifically, it contains 15 phosphorothioate (PS) linkages out of a total of 20 internucleotide linkages, as well as 10 2′-O-methoxyethyl (2′-MOE) modifications. The latter modification is characteristic of antisense oligonucleotides.

**Figure 6:**
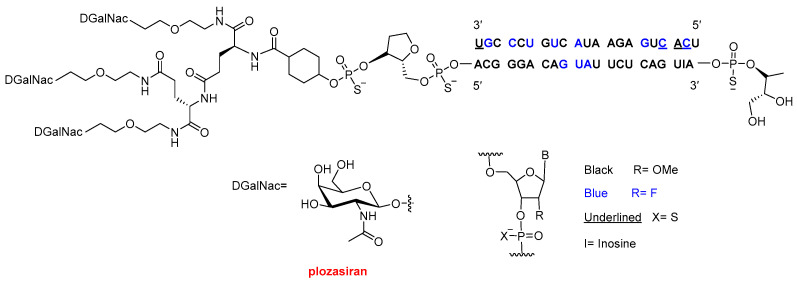
Structure of Redemplo^TM^ (plozasiran).

The third oligonucleotide approved this year, Redemplo^TM^ (plozasiran) (Figure 6), is an siRNA indicated for the treatment of familial chylomicronemia syndrome (FCS). FCS is a rare genetic disorder characterized by extremely elevated triglyceride levels, which can lead to recurrent pancreatitis and the development of xanthomas (fatty deposits in the skin). Redemplo^TM^ is conjugated to three N-acetylgalactosamine (GalNAc) moieties; however, unlike Dawnzera^TM^ and Qfitlia^TM^, these are not arranged in a dendrimeric structure but are instead attached via a di-lysine linker. As with the other oligonucleotides described above, Redemplo^TM^ incorporates multiple chemical modifications to enhance stability, including seven phosphorothioate (PS) linkages, 11 2′-fluoro (2′-F) substitutions, and 31 2′-O-methyl (2′-OMe) substitutions.

**Figure 7:**
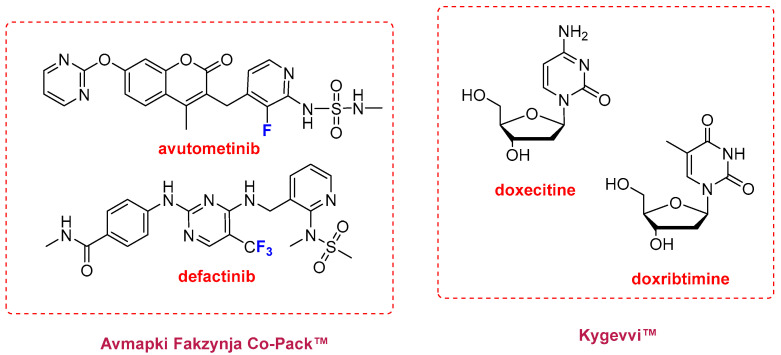
Structures of the combination therapies: Avmapki Fakzynja Co-Pack^TM^ (avutometinib and defactinib) and Kygevvi^TM^ (doxecitine and doxribtimine). Blue color in all figures denotes **F** atom.

In addition to Keytruda Qlex^TM^ (pembrolizumab/berahyaluronidase alfa), two fixed-dose combination therapies were approved in 2025 (Figure 7). Avmapki Fakzynja Co-Pack^TM^, which combines the kinase inhibitors avutometinib and defactinib, was approved for the treatment of ovarian cancer. Both compounds were previously investigated independently as anticancer agents, avutometinib by Chugai Pharmaceutical Co. and defactinib by Pfizer; however, development of defactinib was discontinued in 2015 following a Phase II clinical trial. Kygevvi^TM^, a combination of the pyrimidine nucleosides doxecitine and doxribtimine, was approved for the treatment of thymidine kinase 2 deficiency. This is an ultra-rare genetic disorder, with approximately 120 diagnosed patients worldwide, characterized primarily by muscle weakness and respiratory failure.

Although these two nucleosides underscore the continued importance of natural products as a source of inspiration for drug development, no additional drugs approved this year, beyond these and biologics, can be directly traced to natural product origins.

Drugs containing fluorine atoms are the most represented class in this annual analysis. This year, in addition to Datroway^TM^, the oligonucleotides Qfitlia^TM^ and Redemplo^TM^, and the two components of the Avmapki Fakzynja Co-Pack^TM^ (avutometinib and defactinib), there are 13 additional drugs that contain this pharmaceutically relevant atom. In total, 17 out of 46 drugs approved by the FDA in 2025 (more than one-third) contain one or more fluorine atoms [5]. As discussed in Section 3, the synthesis and commercialization of these compounds may pose environmental concerns, as some of them could be classified as PFAS (per- and polyfluoroalkyl substances), whose use is increasingly recommended to be restricted [6]. All of these fluorine-containing compounds are clear representatives of the small-molecule drugs, typically characterized by relatively extended structures with multiple nitrogen-containing aromatic moieties bearing fluorine substituents.

**Figure 8:**
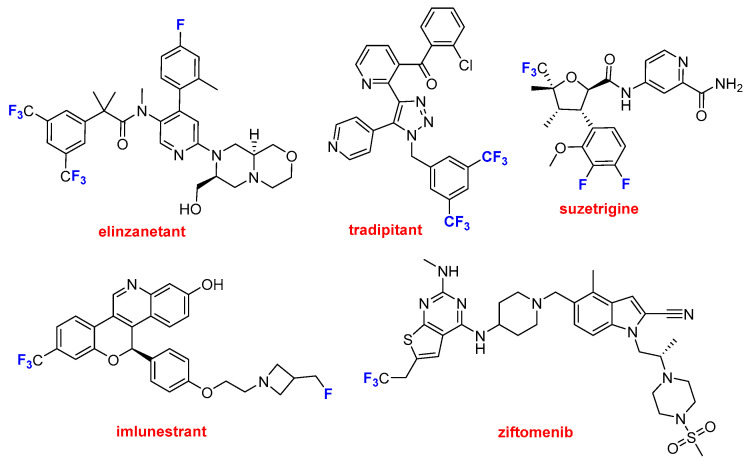
Structure of drugs containing CF_3_ moieties: Lynkuet^TM^ (elinzanetant); Nereus^TM^ (tradipitant); Journavx^TM^ (suzetrigine); Inluriyo^TM^ (imlunestrant); and Komzifti^TM^ (ziftomenib).

This year, five of these drugs contain the CF_3_ moiety (Figure 8). Lynkuet^TM^ (elinzanetant) and Nereus^TM^ (tradipitant) contain two CF_3_ groups. The first one is indicated to modulate neuronal activity associated with thermoregulation and hot flashes during menopause. Nereus^TM^ was approved for the treatment of motion sickness. Journavx^TM^ (suzetrigine), Inluriyo^TM^ (imlunestrant), and Komzifti^TM^ (ziftomenib) contain just one CF_3_ group in addition to other single fluoride atoms on the first three. The first one may be considered as a new class of pain suppressor drug because it manages pain as an opioid but without the risks of addiction. Inluriyo^TM^ is taken orally and is recommended for advanced or metastatic breast cancer with ER-positive, HER2-negative, and ESR1-mutated. Komzifti^TM^ is an anticancer drug used for the treatment of acute myeloid leukemia.

**Figure 9:**
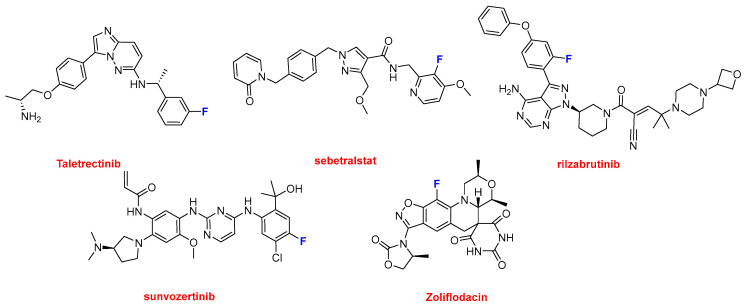
Structure of drugs containing a single aromatic fluorine atom: Ibtrozi^TM^ (taletrectinib); Zegfrovy^TM^ (sunvozertinib); Ekterly^TM^ (sebetralstat); Wayrilz^TM^ (rilzabrutinib); and Nuzolvence^TM^ (zoliflodacin).

Ibtrozi^TM^ (taletrectinib) and Zegfrovy^TM^ (sunvozertinib), each containing a single aromatic fluorine atom, are indicated for the treatment of advanced or metastatic non-small cell lung cancer (NSCLC). These approvals added to the two antibody–drug conjugates (Datroway^TM^ and Emrelis^TM^) already indicated for NSCLC and two more small molecules, Hernexeos^TM^ (zongertinib) and Hyrnuo^TM^ (sevabertinib) (see below) for the same target, underscoring that lung cancer remains one of the most challenging and unresolved malignancies. Ekterly^TM^ (sebetralstat), also with one fluorine atom, was approved for the treatment of hereditary angioedema (HAE), alongside the monoclonal antibody Andembry^TM^ and the oligonucleotide Dawnzera^TM^ (see above). After NSCLC, HAE emerged as the second most targeted disorder in 2025. Also containing a single fluorine atom, Wayrilz^TM^ (rilzabrutinib) is an anticancer agent approved for the treatment of immune thrombocytopenia (ITP), a disorder characterized by immune-mediated platelet destruction. Nuzolvence^TM^ (zoliflodacin) is an antibiotic indicated for the treatment of antibiotic-resistant Neisseria gonorrhoeae (gonorrhea). Notably, it was developed through a public–private partnership between Innoviva Specialty Therapeutics and the Global Antibiotic Research and Development Partnership (GARDP) (Figure 9).

**Figure 10:**
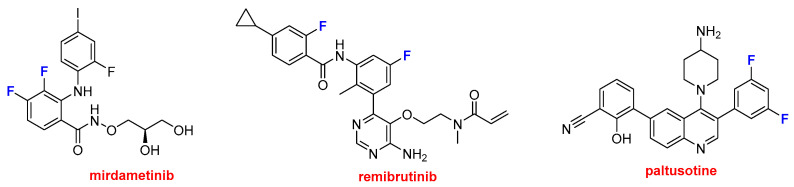
Structure of drugs containing two or more fluorine aromatic atoms: Gomekli^TM^ (mirdametinib); Rhapsido^TM^ (remibrutinib); and Palsonify^TM^ (paltusotine).

Three drugs containing two or more fluorine aromatic atoms were also approved (Figure 10). Gomekli^TM^ (mirdametinib) is recommended for the treatment of neurofibromatosis, a condition characterized by the development of tumors in nerves, the brain, the spinal cord, and the skin. Rhapsido^TM^ (remibrutinib) is indicated for chronic urticaria. Finally, Palsonify^TM^ (paltusotine) is approved for the treatment of acromegaly, a rare disease often caused by a benign pituitary tumor, which leads to progressive enlargement of the hands, feet, facial features, and internal organs.

N-aromatic moieties remain a ubiquitous structural feature of marketed drugs, and 2025 is no exception. This year, 12 of the 13 fluorine-containing drugs include N-aromatic moieties, as do the four components of the combination therapies Avmapki Fakzynja Co-Pack^TM^ (avutometinib and defactinib) and Kygevvi^TM^ (doxecitine and doxribtimine). Even the three oligonucleotides should be included in this category.

In addition to the previously mentioned compounds, 10 more drugs fall into this category. Thus, overall, 27 of the 46 drugs approved in 2025 (more than half) contain N-aromatic moieties. Five of the 10 N-aromatic drugs contain an aminopyrimidine moiety, a structural motif also present in four fluorine-containing drugs, Zegfrovy^TM^ (sunvozertinib), Wayrilz^TM^ (rilzabrutinib), Rhapsido^TM^ (remibrutinib); and Komzifti^TM^ (ziftomenib); as well as in defactinib, one of the APIs in Avmapki Fakzynja Co-Pack^TM^; in the two nucleosides doxecitine and doxribtimine of Kygevvi^TM^; and, notably, in all three oligonucleotides. Overall, 14 of the 44 drugs approved in 2025 (nearly one-third) contain an aminopyrimidine moiety.

**Figure 11:**
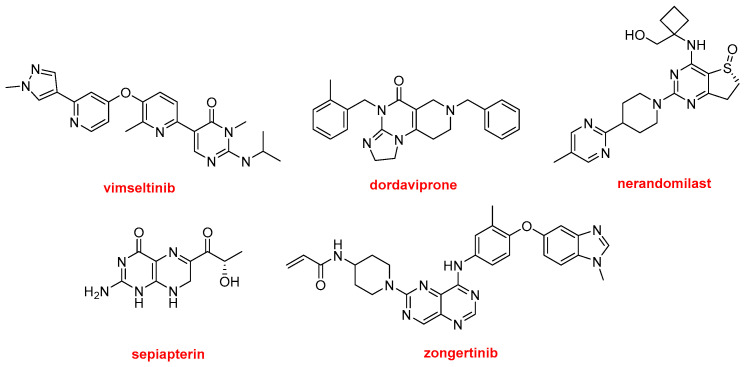
Structure of the N-aromatic drugs containing an aminopyridine moiety: Romvimza^TM^ (vimseltinib); Modeyso^TM^ (dordaviprone); Jascayd^TM^ (nerandomilast); Sephience^TM^ (sepiapterin); and Hernexeos^TM^ (zongertinib).

The five N-aromatic drugs containing an aminopyridine moiety (not cited above) are the following (Figure 11): Romvimza^TM^ (vimseltinib) is indicated for the treatment of tenosynovial giant cell tumor. Modeyso^TM^ (dordaviprone) is approved for the treatment of diffuse midline glioma. Jascayd^TM^ (nerandomilast) is recommended for the treatment of idiopathic pulmonary fibrosis and progressive pulmonary fibrosis. Sephience^TM^ (sepiapterin) is approved for hyperphenylalaninemia, a genetic metabolic disorder in which phenylalanine is not properly metabolized, potentially leading to brain damage, intellectual disability, and seizures. Finally, Hernexeos^TM^ (zongertinib) is indicated for unresectable or metastatic non-squamous non-small cell lung cancer (NSCLC).

**Figure 12:**
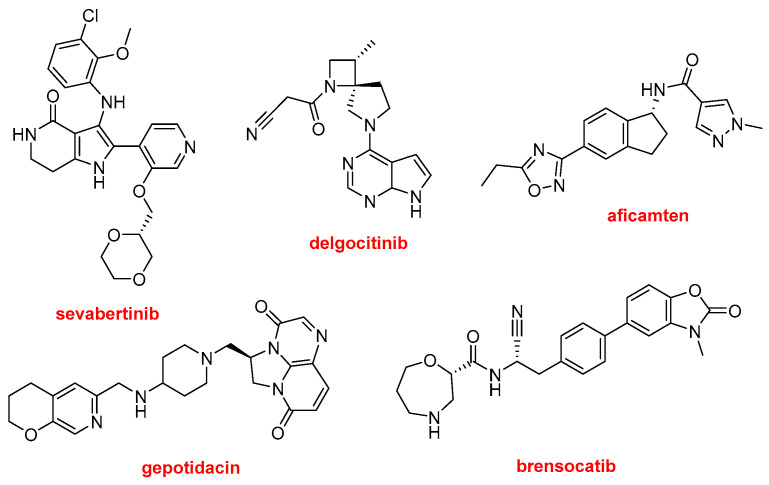
Structure of the N-aromatic drugs not containing fluorine atoms: Hyrnuo^TM^ (sevabertinib); Anzupgo^TM^ (delgocitinib); Myqorzo^TM^ (aficamten); Blujepa^TM^ (gepotidacin); and Brinsupri^TM^ (brensocatib).

Other N-aromatic drugs are the following (Figure 12). Hyrnuo^TM^ (sevabertinib) has also been approved for the treatment of NSCLC. Anzupgo^TM^ (delgocitinib) is recommended for the treatment of autoimmune disorders and hypersensitivity conditions, including inflammatory skin diseases. Myqorzo^TM^ (aficamten) is indicated for patients with symptomatic obstructive hypertrophic cardiomyopathy, where it improves functional capacity and alleviates symptoms. Blujepa^TM^ (gepotidacin) is an antibacterial type II topoisomerase inhibitor approved for the treatment of uncomplicated urinary tract infections in women caused by *Escherichia coli*, *Klebsiella pneumoniae*, *Citrobacter freundii complex*, *Staphylococcus saprophyticus*, and *Enterococcus faecalis*; it is also in use for the treatment of other bacterial infections. Finally, in this section of N-aromatic-based drugs, Brinsupri^TM^ (brensocatib) is approved for the treatment of non–cystic fibrosis bronchiectasis.

**Figure 13:**
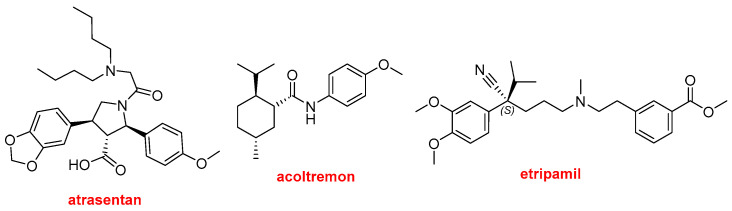
Structure of aromatic drugs Vanrafia^TM^ (atrasentan); Tryptyr^TM^ (acoltremon); and Cardamyst^TM^ (etripamil).

Three more drugs can be classified as aromatic compounds (Figure 13). Interestingly, the three contain alkoxy electron-donating groups. Vanrafia^TM^ (atrasentan) was approved for the reduction of proteinuria. This is the second drug approved in 2025 for proteinuria treatment, together with the mAb, Voyxact^TM^ (sibeprenlimab-szsiis). Tryptyr^TM^ (acoltremon) is a medication used to treat dry eye syndrome. Cardamyst^TM^ (etripamil) for the treatment of episodes of paroxysmal supraventricular tachycardia.

**Figure 14:**
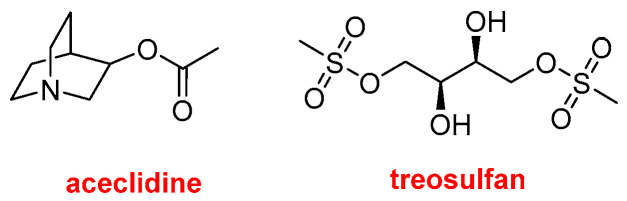
Structure of Vizz^TM^ (aceclidine) and GRAFAPEX^TM^ (treosulfan).

Two drugs with relatively simple chemical structures that do not fall into the previously discussed categories are Vizz^TM^ (aceclidine) and GRAFAPEX^TM^ (treosulfan) (Figure 14). The former, 3-acetoxyquinuclidine, is indicated for the treatment of presbyopia, also known as age-related farsightedness or eye fatigue. GRAFAPEX^TM^ (treosulfan) is L-threitol-1,4-dimethanesulfonate; as suggested by its chemical structure, it functions as an alkylating agent. It is used in combination with fludarabine as a conditioning regimen prior to hematopoietic stem cell transplantation. Fludarabine is a purine analog antineoplastic agent that was approved by the FDA in 1991.

## 3. Conclusions and Perspectives

After five years during which the term COVID dominated biomedical research, 2025 marks the first year in which COVID-19 has become one of many human diseases rather than the central focus of global attention. Even conditions associated with so-called long COVID, characterized by persistent or relapsing symptoms that can significantly impair daily and professional activities, have shown a decline in prominence. Nevertheless, it remains essential not to lower vigilance toward communicable diseases and, in particular, to increase both financial and human investment in understanding and treating long COVID, which is often regarded as a secondary yet enduring consequence of the pandemic.

Although somewhat removed from traditional drug discovery, gene therapy, most notably CRISPR-based technologies, continues to hold significant promise for personalized medicine, with applications in blood disorders, cancer immunotherapy, infectious diseases, and neurodegenerative conditions [7]. While the transformative potential of CRISPR is unquestionable, significant challenges remain, particularly those related to off-target effects and ethical considerations.

Although only one peptide-based drug was approved in 2025, peptide therapeutics, particularly those targeting diabetes and obesity, such as agonists of glucagon-like peptide-1 (GLP-1) and glucose-dependent insulinotropic polypeptide (GIP), continue to dominate the pharmaceutical landscape. Market analysts project that these therapies could reach a global market value of USD 100–150 billion by 2030, with approximately 9% of the U.S. population expected to be receiving such treatments by that time [8,9].

The growing demand for large quantities of peptides, typically 30–40 amino acids in length, is reshaping peptide manufacturing. For decades, peptides have been synthesized predominantly using solid-phase peptide synthesis (SPPS), pioneered by Merrifield. However, SPPS relies on a solid polymeric support that occupies substantial reactor volume, thereby limiting batch productivity; requires large excesses of reagents; and, most critically, consumes vast amounts of solvent, rendering it a poorly sustainable synthetic approach.

These limitations have driven renewed interest in tag-assisted liquid-phase peptide synthesis (TA-LPPS) [10]. Like SPPS, TA-LPPS is a continuous process in which intermediates are not isolated; however, the chemistry occurs entirely in solution, with the solid support replaced by a hydrophobic tag. This tag enables efficient phase separation during extraction, allowing the growing peptide chain to be readily separated from excess reagents and by-products. From a sustainability perspective, the advantages are substantial: the process mass intensity (PMI) for SPPS is approximately 13,000 (i.e., 13,000 kg of reagents and solvents per kilogram of peptide produced), whereas the PMI for a TA-LPPS process is close to 1000, representing a dramatic improvement.

As outlined earlier, one of the major challenges facing the chemical and pharmaceutical industries is the reduction or elimination of PFAS. The C–F bond is exceptionally strong and highly resistant to degradation, leading to persistent contamination of soil and water. In peptide synthesis, the final deprotection step, which includes cleaving the peptide from the resin in SPPS, traditionally relies on trifluoroacetic acid (TFA). Minimizing or avoiding the use of TFA is therefore critical. Accordingly, alternative and less harmful reagents, such as FeCl_3_/acetic acid [11] and methanesulfonic acid (MSA)/formic acid [12], are increasingly being explored for this purpose. Nevertheless, fluorine remains a key motif in pharmaceutical design, as the presence of C–F and CF_3_ groups in active pharmaceutical ingredients often confers enhanced biological activity that cannot be readily achieved by substitution with hydrogen or other atoms. The presence of fluorine-containing drugs remains an unsolved challenge from a sustainability point of view.

**Figure 15:**
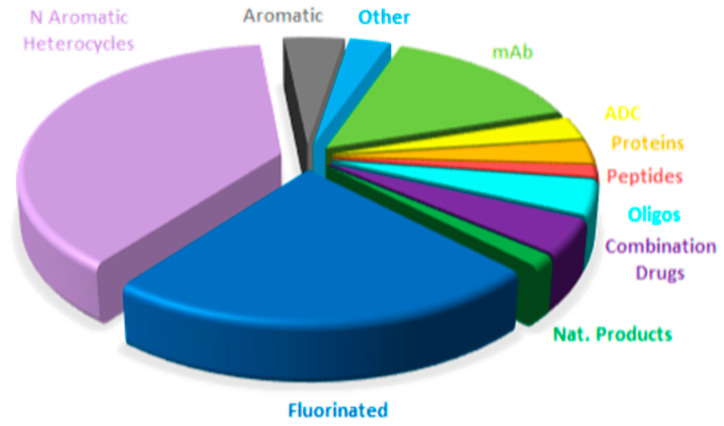
Drugs approved by the FDA in 2025 classified on the basis of chemical structure (drugs can belong to more than one class).

Figure 15 shows drugs approved by the FDA in 2025 classified on the basis of chemical structure (drugs can belong to more than one class).

In terms of new drug approvals (46), 2025 has been a good year, slightly below the totals of previous years [50 in 2024, 55 in 2023, 37 in 2022, 50 in 2021, 53 in 2020, 48 in 2019, and 59 in 2018]. This minor decline is likely attributable to political factors in Washington rather than any weakness in the pharmaceutical industry.

Biologics accounted for 12 approvals, slightly fewer than in recent years, but still representing 25% of all drugs approved in 2025. Among these, 11 monoclonal antibodies (mAbs), eight as single agents, one in combination with a protein, and two as components of ADCs, remain the most representative class of drugs. After a two-year period without new ADC approvals, two were authorized in 2025, bringing the total number of ADCs on the market to 16. It is noteworthy that the first FDA-approved mAb was muromonab-CD3 (Orthoclone OKT3^TM^) in 1986, and the first ADC, gemtuzumab ozogamicin (Mylotarg^TM^), was approved in 2000 (withdrawn in 2010 and reapproved in 2017), highlighting the substantial advancements in chemical and biotechnological industries.

For biologics, cancer remains the primary therapeutic target, consistent with the overall trend for newly approved drugs. However, new indications continue to emerge each year, underscoring the pharmaceutical industry’s innovative capacity.

TIDES, comprising three oligonucleotides and one peptide this year, represents nearly 10% of all drugs approved in 2025, consistent with recent trends. The three oligonucleotides (two siRNAs and one antisense oligonucleotide, ASO) all feature three copies of N-acetylgalactosamine (GalNAc), widely recognized as the gold standard for liver-targeted delivery. Given the growing impact of oligonucleotide therapeutics, it may be time to acknowledge the contributions of oligonucleotide chemistry at the level of the Nobel Prize.

Once again, TIDES and biologics together account for one-third of all drugs approved this year (15 out of 46), reaffirming the slight decrease in the small molecules in the pharmaceutical market, which previously dominated.

In addition to biologics and TIDES, the two components (doxecitine and doxribtimine) of Kygevvi^TM^ find their roots in natural products.

Two antibiotics have been approved: Zoliflodacin^TM^ (nuzolvence) for the treatment of gonorrhea, the most prevalent sexually transmitted disease, and Gepotidacin^TM^ (blujepa) for urinary tract infections. After many years of limited investment in antibiotic research, the market is now beginning to reap the benefits of a renewed research surge. Consistent with this trend, three antibiotics were approved in the previous year (2024).

When only small-molecule drugs are considered, excluding TIDES and biologics, nearly three-quarters (23 of 30) feature N-aromatic moieties and fluorine atoms. This proportion is the same as that observed in the previous year, reaffirming the prevalence of these structural motifs in drug discovery.

**Figure 16:**
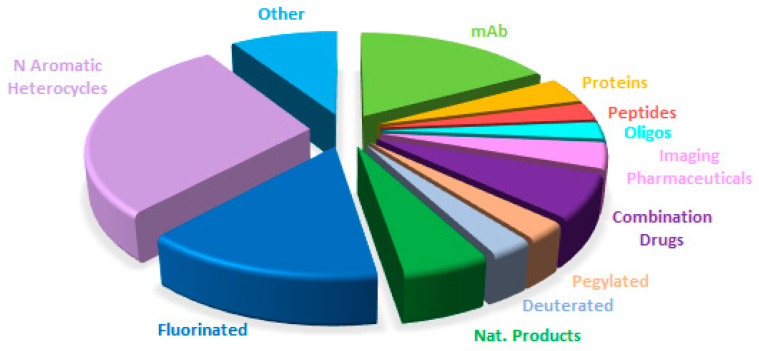
Similarly to Figure 15 for 2024, adapted with permission from ref. [1]. Copyright 2024, copyright MDPI.

**Figure 17:**
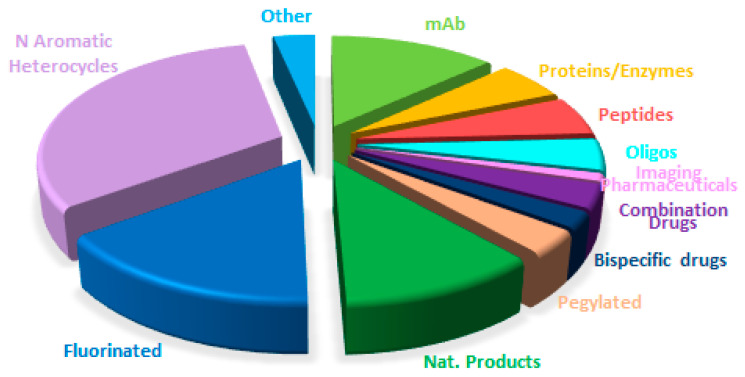
Similarly to Figure 15 for 2023, taken with permission from ref. [13]. Copyright 2024, copyright MDPI.

**Figure 18:**
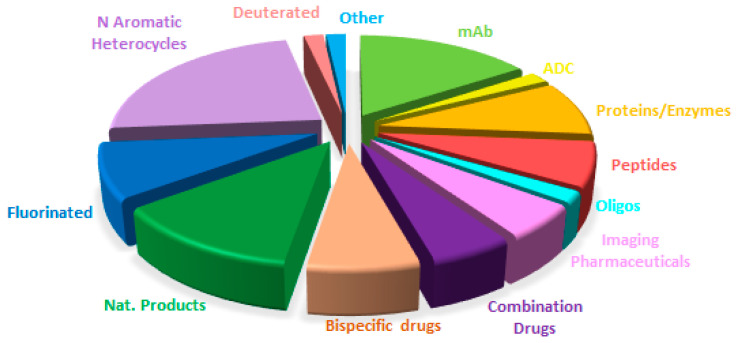
Similarly to Figure 15 for 2022, taken with permission from ref. [14]. Copyright 2023, MDPI.

A comparison of drug distribution across different classes shows that overall trends observed in recent years are largely maintained, with a few exceptions (Figure 15, Figure 16, Figure 17 and Figure 18). For example, the number of drugs inspired by natural products is lower in 2025, and no new imaging pharmaceuticals, pegylated compounds, or deuterated drugs were approved. Nevertheless, the major long-term trends remain consistent, reflecting a consolidation of biologics and TIDES occurring at the expense of small molecules.

As in previous years, cancer, rare diseases, and skin and infectious diseases remain the primary indications for drugs approved by the FDA in 2025. Five drugs are indicated for non-small cell lung cancer (NSCLC), including two ADCs [Datroway^TM^ (datopotamab deruxtecan) and Emrelis^TM^ (telisotuzumab vedotin-tllv)] and four small molecules [Ibtrozi^TM^ (taletrectinib), Zegfrovy^TM^ (sunvozertinib), Hernexeos^TM^ (zongertinib), and Hyrnuo^TM^ (sevabertinib)]. Hereditary angioedema (HAE) is addressed by three newly approved therapies: the monoclonal antibody Andembry^TM^ (garadacimab-gxii), the oligonucleotide Dawnzera^TM^ (donidalorsen), and the small molecule Ekterly^TM^ (sebetralstat). In addition, two drugs were approved for the reduction of proteinuria: the monoclonal antibody Voyxact^TM^ (ibeprenlimab-szsi) and the small molecule Vanrafia^TM^ (atrasentan).

Collectively, these indications highlight a fundamental principle of modern drug discovery: *There Is More Than One Way To Skin a Cat or There Is More Than One Way to achieve therapeutic success for a given disease.*

We confer the honorific title of “2025 Drug of the Year” on Forzinity^TM^ (elamipretide) in recognition of its elegant simplicity and clinical significance. Elamipretide is a small tetrapeptide containing a D-arginine residue and a dimethylated tyrosine, and it represents the first approved therapy to improve muscle strength in patients with Barth syndrome (BTHS), a rare, life-threatening condition that can lead to premature death.

The pharmaceutical industry continues to exhibit robust growth, fueled by substantial investments across all stages, particularly in drug delivery systems, medical devices, and artificial intelligence (AI).

There is no doubt that AI is already having a fully transformational impact on drug discovery. Although one clear impact will be the reduction of time and cost (currently, 10-15 years and an average of $2 billion), AI is expected to have a more significant impact in developing safer and more effective drugs for treating various diseases through the discovery of new targets. In this regard, it is important to have in mind the efforts of different governmental institutions, such as the National Institutes of Health (NIH), the Chinese government, and the European Commission, among others, funding research projects, formulating policies, and strengthening international cooperation in the binomial AI and drug discovery [15]. In peptide-based drug discovery, due to the intrinsic characteristics of amino acids (acidic, basic, hydrophobic, hydrophilic, and interchangeable) as peptide moieties, the impact of AI is likely to be one of the first to be noted [16]. This is highlighted by the effort of de la Fuente-Nunez and co-workers to propose new/old antimicrobial peptides from the archaeal proteome [17].

As we conclude this annual report, it is important to draw attention to the escalating costs of pharmaceuticals, particularly those targeting chronic diseases. While these therapies often represent major scientific breakthroughs, their high prices mean that they are accessible to only a limited portion of the global population, exacerbating existing health inequalities. The issue of access is especially acute in low- and middle-income countries, where many patients cannot afford these life-changing treatments. As highlighted by the Access to Medicine Foundation, the pharmaceutical industry continues to face challenges in making new drugs widely available, underscoring the need for innovative pricing models, global partnerships, and policy interventions that can ensure equitable access. Addressing these challenges is critical not only for improving health outcomes but also for fulfilling the broader social responsibility of the industry in promoting global health equity [18].

One year ago, we questioned the potential impact of the new U.S. administration on the drug discovery process. Our initial impression suggests a slight slowdown, though this trend will need to be confirmed over the coming years.

## Figures and Tables

**Table 1:** Biologics approved by the FDA in 2025 [2].

Trade Name ^a^	Active Ingredient ^a^	Class	Indication
Andembry^TM^	Garadacimab-gxii	mAb	Hereditary Angioedema (HAE) (swelling of skin or mucous membranes)
Imaavy^TM^	Nipocalimab	mAb	Myasthenia Gravis (chronic autoimmune disease resulting in muscle weakness)
Enflonsia^TM^	Clesrovimab-cfor	mAb	To prevent respiratory syncytial virus (RSV)
Exdensur^TM^	Depemokimab	mAb	Asthma
Lynozyfic^TM^	Linvoseltamab	mAb	Relapsed/refractory Multiple Myeloma
Penpulimab-kcqx	Penpulimab-kcqx	mAb	Recurrent/metastatic Non-Keratinizing Nasopharyngeal Carcinoma (NPC)
Voyxact^TM^	Sibeprenlimab-szsi	mAb	Reduction of proteinuria in patients with Immunoglobulin A nephropathy
Yartemlea^TM^	Narsoplimab-wuug	mAb	Treatment of thrombotic microangiopathy associated with hematopoietic stem cell transplantation.
Datroway^TM^	Datopotamab deruxtecan	ADC	Unresectable/metastatic, HR-positive, HER2-negative, Breast Cancer and Non-small Cell Lung Cancer (NSCLC)
Emrelis^TM^	Telisotuzumab vedotin-tllv	ADC	Advanced/metastatic, non-Squamous, NSCLC
Keytruda Qlex^TM^	Pembrolizumab/ Berahyaluronidase alfa	mAb/enzyme	Broad spectra of solid tumors
Lerochol^TM^	Lerodalcibep-liga	Fusion protein	Hypercholesterolemia

^a^ Trade name used in the U.S.

## Data Availability

No new data were created or analyzed in this study. Data sharing is not applicable.

## References

[1] de la Torre B.G., Albericio F. (2025). The Pharmaceutical Industry in 2024. An Analysis of FDA Drug Approvals from the Perspective of Molecules. Molecules.

[2] U.S. Food and Drug Administration (FDA) https://www.fda.gov/drugs/novel-drug-approvals-fda/novel-drug-approvals-2025.

[3] U.S. Food and Drug Administration (FDA) https://www.fda.gov/vaccines-blood-biologics/development-approval-process-cber/2025-biological-license-application-approvals.

[4] https://peptherdia.herokuapp.com/.

[5] Shabir G., Saeed S., Zahid W., Naseer F., Riaz Z., Khalil N., Muneeba, Albericio F. (2023). Chemistry and Pharmacology of Fluorinated Drugs Approved by FDA (2016–2022). Pharmaceuticals.

[6] https://echa.europa.eu/hot-topics/perfluoroalkyl-chemicals-pfas.

[7] Xu Y., Li Z. (2020). CRISPR-Cas systems: Overview, innovations and applications in human disease research and gene therapy. Comput. Struct. Biotechnol. J..

[8] Morgan Stanley Research (2025). The Exponential Growth of Obesity Drugs. https://www.morganstanley.com/insights/articles/weight-loss-medication-market-unstoppable-growth.

[9] McKinsey & Company (2025). What Are GLP-1 Medications?. https://www.mckinsey.com/featured-insights/mckinsey-explainers/what-are-glp-1-medications.

[10] Sharma A., Kumar A., de la Torre B.G., Albericio F. (2022). Liquid Phase Peptide Synthesis (LPPS), a Third Wave for the Preparation of Peptides. Chem. Rev..

[11] Pawlas J., André C., Rasmussen J.H., Ludemann-Hombourger O. (2024). Brønsted Acid–Lewis Acid (BA–LA) Induced Final Deprotection/Peptide Resin Cleavage in Fmoc/t-Bu Solid-Phase Peptide Synthesis: HCl/FeCl_3_ and AcOH/FeCl_3_ as Viable PFAS-Free Alternatives for TFA. Org. Lett..

[12] Fidha F., Kumar A., Leko M., Marder O., Burov S., Sharma A., de la Torre B.G., Albericio F. (2020). Methanesulfonic Acid (MSA) in Solid-Phase Peptide Synthesis: A Sustainable Option for Global Deprotection. Green Chem..

[13] de la Torre B.G., Albericio F. (2024). The Pharmaceutical Industry in 2023. An Analysis of FDA Drug Approvals from the Perspective of Molecules. Molecules.

[14] de la Torre B.G., Albericio F. (2023). The Pharmaceutical Industry in 2022. An Analysis of FDA Drug Approvals from the Perspective of Molecules. Molecules.

[15] Li C. (2025). AI alignment is all your need for future drug discovery. Front. Artif. Intell..

[16] Roque-Borda C., Pavan F., de la Torre B.G., Albericio F. (2026). Redefining peptide chemistry beyond accumulating analogues. Nat. Rev. Chem..

[17] Torres M.D.T., Wan F., de la Fuente-Nunez C. (2025). Deep learning reveals antibiotics in the archaeal proteome. Nat. Microbiol..

[18] Access to Medicine Foundation. https://accesstomedicinefoundation.org/.

